# Dimercaprol (BAL): Insights into conformational stability, fragmentation pathways via tandem LR-ESI, HR-EI mass spectrometry, and gas-phase thermochemical properties from quantum chemical calculations

**DOI:** 10.1371/journal.pone.0349950

**Published:** 2026-06-01

**Authors:** Miguel Fernando Molano, Alba Marcela Gómez Gómez, Alejandro Moncayo-Lasso, Carlos A. Bejarano, John Edward Diaz, Cristian Buendía-Atencio, Miguel Ángel Delgado, Vaneza Paola Lorett Velásquez, Alix E. Loaiza, Sol M. Mejía

**Affiliations:** 1 Departamento de Química, Facultad de Ciencias, Universidad Antonio Nariño, Bogotá, Colombia; 2 Facultad de Ciencias y Educación, Universidad Distrital Francisco José de Caldas, Bogotá, Colombia; 3 Facultad de Medicina y Ciencias de la Salud, Grupo de Investigación sobre Agentes Radiológicos, Biológicos y Químicos, Universidad Militar Nueva Granada, Bogotá, Colombia; 4 Departamento de Química, Facultad de Ciencias, Pontificia Universidad Javeriana, Bogotá, Colombia; University of Botswana, BOTSWANA

## Abstract

Dimercaprol (British antilewisite, BAL) is a long-established chelating agent used in the treatment of heavy metal poisoning; however, its physicochemical and thermochemical properties have not yet been fully characterized. In this study, we combined gas-phase quantum chemical calculations with high-resolution mass spectrometry to investigate the conformational stability, fragmentation pathways, and thermochemical parameters of BAL. Fragmentation behavior was examined by gas chromatography/mass spectrometry-QTOF under electronic ionization conditions, and the resulting spectra were interpreted through proposed dissociation pathways involving water, hydrogen sulfide, and thiyl radical losses, supported by reaction enthalpies calculated at the theoretical level M06-2X/6–311++G(*3df,3pd*). Conformational analysis identified five low-energy structures (BAL-1 to BAL-5), where intramolecular hydrogen bonds and gauche/anti interactions play a key role in stability; BAL-3 was consistently predicted as the lowest-energy conformer. Vibrational frequencies calculated with the B3LYP, M06-2X, and MN15 functionals showed good agreement with experimental FTIR and Raman data. The thermochemical properties were further evaluated using G*n* composite methods (G3MP2B3, G3B3, G4MP2 and G4) which yielded an average standard enthalpy of formation at ΔH°_(*f,298K*)_ of –45.6 ± 1.1 kcal/mol. This work provides a detailed experimental and theoretical characterization of dimercaprol, providing information on its possible fragmentation mechanism and conformational landscape, and offering a thermochemical framework that could support future pharmacological, toxicological and environmental applications.

## 1. Introduction

Heavy metal poisoning is a major public health problem affecting people of all ages worldwide. According to the World Health Organization (WHO) [1], this problem is common in both developing and industrialized countries, where environmental pollution can trigger serious illnesses due to prolonged exposure to highly toxic metals. The accumulation of these elements in the human body can lead to a range of symptoms, from headaches and nausea to irreversible brain and kidney damage [2–4].

During World War II, the University of Oxford developed an antidote known as British Anti-Lewisite (BAL, 2,3-dimercaptopropanol, C_3_H_8_OS_2_), a drug initially designed to treat poisoning by arsenic, gold, and mercury [5]. Its synthesis, first described by Stocken and Thompson [6], involves bromination of an allylic alcohol to produce glycerol dibromohydrin, followed by controlled reaction with sodium hydrosulfide. Early investigations were based on the hypothesis that the products of the reaction between arsenic and sulfhydryl (–SH) groups were less toxic than free arsenic [7–9]. Among the compounds evaluated, BAL proved to be the most effective and least toxic. Today, dimercaprol remains widely used in pharmacology due to its ability to form highly soluble chelates, which facilitate the urinary excretion of heavy metals [10,11].

BAL was also used to treat Wilson’s disease (hepatolenticular degeneration), a disorder characterized by excessive copper accumulation in the liver and brain. However, its clinical use revealed significant intrinsic toxicity: approximately 3% of treated children developed acute kidney injury, and about 13% experienced nephrotoxicity. Hypertension, tachycardia, chest pain, nausea, vomiting, and abdominal pain were also reported, among other adverse effects [12–14]. Experimental toxicology data show that dimercaprol exhibits a narrow therapeutic margin, with an intraperitoneal LD50 of approximately 44 mg·kg-1 in mice and 28–40 mg·kg-1 in rats, while doses exceeding 5 mg·kg-1 in humans can cause pronounced cardiorespiratory distress and hepatic dysfunction [15,16]. Toxicity is further exacerbated when BAL is administered intravenously or in the presence of lipid-soluble solvents, emphasizing its classification as a toxic chelating agent in clinical use and motivating the development of safer analogs such as dimercaptosuccinic acid (DMSA) and dimercaptopropanesulfonic acid (DMPS) [17].

Recent studies have addressed several aspects of the mechanism of action of dimercaprol [18]. It has been suggested that BAL competes with endogenous sulfhydryl groups in tissues, interfering with cellular respiration, displacing essential metal cofactors from metabolic enzymes, and increasing capillary permeability. The metabolic degradation and excretion of BAL are completed within ~4 h, as the drug is metabolized in the liver and eliminated as inactive products in urine. Owing to its lipophilic nature, dimercaprol rapidly penetrates intracellular compartments, accumulating in the liver, kidneys, brain, and small intestine. Consequently, complexes formed with mercury and other metals may redistribute to the central nervous system after treatment [17].

Given that dimercaprol (BAL) exhibits intrinsic toxicity and that its efficacy depends on the integrity of its thiol groups, understanding the reactivity and degradation pathways of its products is crucial. As a secondary thiol, it is susceptible to oxidation and fragmentation mechanisms similar to those observed in poly(thiourethanes) and sulforaphane-based systems [19–21], where the presence of sulfur, although essential for chelation, shows limited stability against thermal agents, UV radiation, and nucleophilic attack by endogenous thiols such as glutathione. In long-chain thiols, aerobic degradation has been shown to begin with the formation of sulfenic acids (–SOH), highly reactive intermediates that can evolve into sulfinic and sulfonic acids or participate in radical-mediated processes leading to disulfides or other irreversible products [22–24]. These transformations can significantly alter the chelating capacity of the BAL, modify its toxicity profile, and generate potentially harmful oxidized or radical species, with both pharmacological and environmental implications that have not yet been fully characterized.

Computational approaches have been widely applied to explore the kinetics and reaction mechanisms of BAL, particularly focusing on the influence of catalytic agents such as water and ammonia [25]. Sahu et al. performed a conformational study using the semi-empirical PM6 method, later refined through M06-2X/TZVP calculations [23], which identified four main conformers. However, their analysis lacked detailed information on dihedral angles, relative energies, and vibrational frequencies, limiting a complete understanding of the conformational landscape.

Further investigations by Rajalakshmi combined theoretical and experimental strategies, employing FTIR and Raman spectroscopy alongside DFT-B3LYP/6–31 + G(*d,p*) calculations to elucidate the molecular and vibrational characteristics of BAL [24]. These efforts significantly contributed to describing its structural and spectroscopic properties but did not fully address the molecule’s conformational diversity or the role of intramolecular interactions that may stabilize specific geometries.

Over the past decade, renewed interest in BAL has emerged due to its therapeutic potential in diverse pathologies. Ran and Riyi (2017) demonstrated its ability to lower acrolein levels—a toxic aldehyde associated with oxidative damage—in a rat model of spinal cord injury, while also protecting PC-12 neuronal cells *in vitro*, as confirmed by NMR spectroscopy [25]. Shi et al. reported that BAL attenuates oxidative stress in animal models of Parkinson’s disease, leading to improvements in motor function and tissue integrity. These findings suggest that BAL could effectively act in conditions linked to lipid peroxidation, including neurodegenerative diseases, traumatic injuries, and certain cancers [26].

In oncology, a 2021 study investigated the use of the BAL–arsenic trioxide (BAL–ATO) complex as a radiosensitizer in a murine pancreatic cancer model. The treatment significantly reduced tumor hypoxia and modulated cancer stem cell markers, resulting in a 73% tumor reduction when combined with radiotherapy [27]. In 2023, Ashwini Sri Hari et al. demonstrated that BAL (also referred to as DMP) increases glutathione (GSH) levels by post-translational activation of glutamate–cysteine ligase (GCL), the rate-limiting enzyme in GSH biosynthesis. In a zebrafish model of Dravet syndrome, BAL treatment not only elevated GSH levels but also reduced seizure activity and improved electrophysiological parameters, likely through modulation of the mTOR–redox pathway [28].

Despite its long history of clinical use, significant toxicity, and growing interest in its therapeutic and environmental applications, dimercaprol still lacks complete physicochemical and thermochemical characterization. Little is known about its fragmentation pathways and the associated thermodynamic parameters that underlie molecular stability, limiting our understanding of its structural behavior and the degradation species that could contribute to adverse effects or environmental persistence.

In this context, the present study integrates gas-phase quantum chemical calculations with gas chromatography–mass spectrometry coupled to a quadrupole time-of-flight analyzer (GC/MS-QTOF) to elucidate the ionic fragmentation pathways of dimercaprol under electron ionization (EI) conditions. These pathways describe the gas-phase ion chemistry of BAL, providing detailed insights into energetic profiles and transient intermediates. The resulting data advance the thermochemical characterization of this compound and contributes to establishing a stronger molecular framework for its potential redesign or application in new scientific contexts.

## 2. Materials and methods

### 2.1. Gas chromatography–high-resolution mass spectrometry (GC/MS-QTOF) analysis

Data acquisition was performed on an Agilent Technologies 7890B gas chromatograph coupled to an Agilent 7250 GC/Q-TOF mass spectrometer, equipped with a split/splitless injection port (250 °C, split ratio 50:1) and an Agilent 7693A automatic injector. Ionization was carried out by electron impact (EI) at 70 eV. Chromatographic separation was achieved using an Agilent J&W HP-5MS capillary column (30 m × 0.25 mm i.d., 0.25 µm film thickness) with helium as carrier gas at a constant flow rate of 0.7 mL/min. [29–32]

The oven temperature was programmed from 60 °C (1 min) to 325 °C (10 min) at a rate of 10 °C/min. The transfer line, ion source, and quadrupole temperatures were maintained at 280 °C, 230 °C, and 150 °C, respectively. Mass spectrometric detection was performed over an m/z range of 50–600 at an acquisition rate of 5 spectra/s.

### 2.2. Quantum chemical calculations

The computational strategy begins with the optimization of the BAL (C_3_H_8_OS_2_) molecule using the B3LYP, M06-2X, and MN15 functionals, combined with the 6–31 + G(*d,p*) base set. The B3LYP functional is often considered the default GGA DFT hybrid functional, offering reliable geometries for many main group molecules, including sulfur-containing heterocycles [33]. M06-2X is a metahybrid functional with 54% Hartree-Fock exchange, which has proven to be a robust alternative given its MUE deviation of 0.74 kcal/mol in ABDE4, making it optimal for predicting reaction energies and barrier heights in dithiol compound chemistry [34].

Furthermore, the MN15 functional was used, which has a 44% Hartree-Fock exchange [35] and offers balanced performance according to Haoyu S. Yu et al. This functional presents a mean error (MUE) of 1.85 kcal/mol and an accuracy in calculating non-covalent interactions (MUE) of 0.25 kcal/mol using the NC87 database. The ability of this functional to model thermochemical properties of sulfur compounds was specifically tested in atomization energies using the S25 test set and electron affinities with an MUE of 1.57 kcal/mol in EA13/03. Therefore, this MN15 functional is appropriate since it belongs to the same family of functionals developed by the Truhlar group to be used in this work [35].

**Fig 1:**
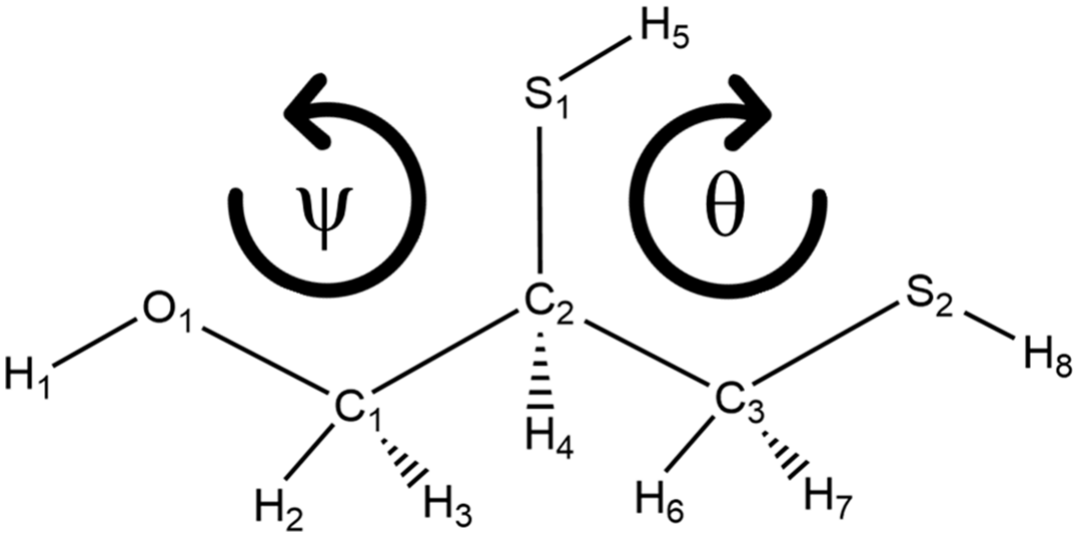
Atom labeling of the BAL (2,3-Dimercaptopropan-1-ol).

Using the obtained geometries, the dihedral angles ψ = O1C1C2S1 and θ = S1C2C3S2 (see Fig 1) were selected to estimate the rotational energy profiles. These angles were varied in 5° increments, while the other structural parameters remained relaxed. The corresponding rotational barrier was calculated as the difference between the highest point on the potential energy surface and the local minimum energy conformer. The geometries at the local minimum energy points were completely reoptimized using the same functionals employed previously, but with an extended basis set, 6–311++G(*3df,3pd*). For the estimation of harmonic vibrational frequencies, second-order derivative analytical methods were used at the same theoretical level mentioned above, and the absence of imaginary frequencies was verified. At the employed levels of theory, the recommended vibrational frequency scaling factors are reported to be very close to unity when using extended triple-ζ basis sets with diffuse and polarization functions; therefore, no additional scaling was applied [36]. The vibrational band assignments were analyzed using the molecular visualization program GaussView and compared with the reported experimental data.

### 2.3. Thermochemistry properties

Calculations to estimate the enthalpy of formation of BAL were performed using the total atomization energy method with high-precision computational thermochemical models, such as G3MP2B3, G3B3, G4MP2 and G4 [37–40]. Since dimercaptopropan-1-ol contains H, C, O and S atoms, the BAC-G3MP2B3 and BAC-G3B3 [37,41] procedures were used, as they have been validated in thermochemical calculations; These protocols apply empirical atomic, molecular, and bond-pair additive corrections (BACs) to the enthalpy of formation values calculated using these methods, compensating for systematic biases in heteroatoms such as sulfur and improving accuracy to ~1 kcal/mol RMS, using the correction equation (see Equation 1) where E_BAC−atom_, E_BAC−molecule_, and E_BAC−bond_ are the contributions of the atomic, molecular, and bond corrections, respectively.


$$\begin{array}{c} E_{BAC-Correction\;(total)}=\;E_{BAC-Atom}+E_{BAC-molecule}+\sum {E\;(A_{i}A_{j})}_{BAC-Bond} \end{array}$$ (1)

Furthermore, the Gn chemical models were modified in their first and second steps (geometries and zero-point energies) using the geometry obtained with the B3LYP/6–311++G(*3df,3pd*) level of theory, which employs a larger basis set than the original method. This modified approach is referred to in this work as G3MP2B3//B3LYP/6–311++G(*3df,3pd*) and G3B3//B3LYP/6–311++G(*3df,3pd).* All calculations were performed using Gaussian 16 software.

Furthermore, this work proposes a set of half-reactions to describe the dissociation pathways of (BAL). Based on experimental results, the protonated precursor ions, ionic fragments, and neutral loss conformations are identified. The reported energies were obtained at the theoretical level M06-2X/6–311++G(*3df,3pd*) for each half-reaction studied [36].

## 3. Results and discussion

### 3.1. Energies for the Dissociation Pathways of BAL

**Scheme 1:**
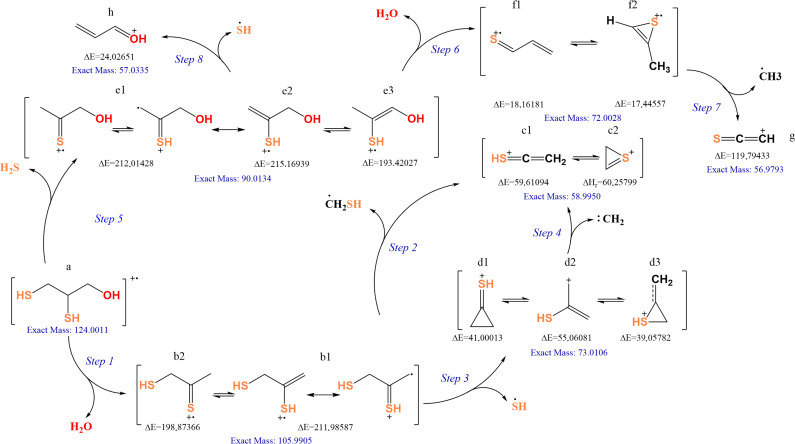
BAL degradation pathway with relative energies ∆E (kcal/mol) calculated with an M06-2X/6-311++G(*3df,3pd*) level of theory.

**Fig 2:**
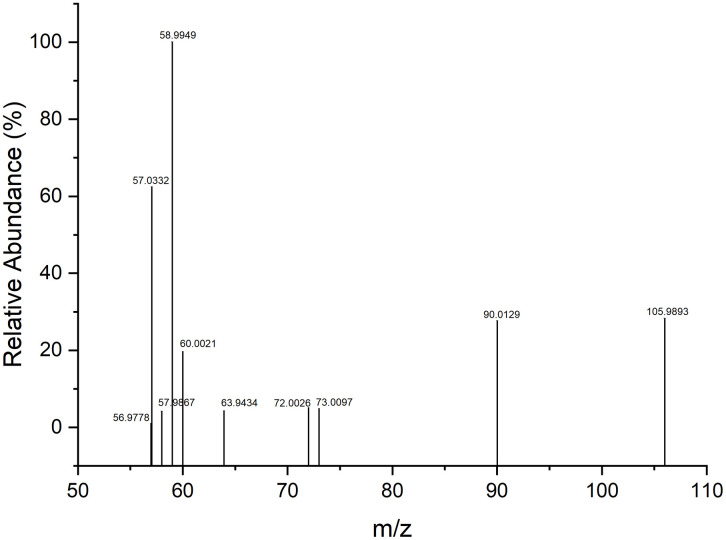
High-resolution mass spectrum obtained for dimercaprol (BAL) by GC/MS-QTOF with electron ionization (EI).

The high-resolution mass spectrum of the BAL radical cation is shown in Fig 2, and the observed fragments are summarized in Table 1. The proposed fragmentation pathway (Scheme 1) involves eight sequential steps starting from the molecular radical cation [C_3_H_8_OS_2_∙⁺]. Relative energies (ΔE) for each step were calculated at the M06-2X/6–311++G(*3df,3pd*) level, this method has been extensively benchmarked for main-group thermochemistry and reaction barrier heights, showing good performance across diverse datasets [34]. These values allow estimation of the relative energies of each fragmentation channel observed under the high-energy conditions of the GC–MS experiment with electron ionization (EI) [42–44]. Although some fragmentation steps exhibit relatively high ΔE values (>100 kcal/mol), it is important to emphasize that electron ionization (EI) is not a thermal process. Under EI conditions (~70 eV), substantial internal energy is deposited into the molecular ion, enabling access to high-energy dissociation channels. Therefore, fragmentation feasibility should be interpreted within a unimolecular dissociation framework governed by internal energy redistribution rather than by classical thermal enthalpic criteria [30,31,45].

**Table 1:** High-resolution experimental peaks and exact masses calculated for dimercaprol-derived fragments (BAL) in GC/MS-QTOF with electron ionization (EI).

Experimental m/z	Molecular Formula
124.0007	C_3_H_8_OS_2_∙^+^
105.9893	C_3_H_6_S_2_∙^+^
90.0129	C_3_H_6_OS∙^+^
73.0097	C_3_H_5_S_2_^+^
72.0026	C_3_H_4_S∙^+^
58.9949	C_2_H_3_S^+^
57.0322	C_3_H_5_O^+^
56.9778	C_2_HS +

Step 1. The molecular ion [C_3_H_8_OS_2_∙⁺] loses H_2_O, producing the fragment [C_3_H_6_OS_2_∙⁺] at m/z 105.99. Two isomeric cations were identified. Pathway angle of 122.78°, whereas pathway **b2** (ΔH*ᵣ* = 198.87 kcal/mol) leads to a radical cation with charge localization preferentially on the sulfur atom.

Step 2. The fragment [C_3_H_5_S_2_∙⁺] undergoes loss of a methanethiol radical (∙CH_2_SH), yielding [C_3_H_5_S_2_∙⁺] at m/z 58.99, the base peak of the spectrum. Energy analysis confirmed that **b1** is the more favorable route, being 13.11 kcal/mol lower than **b2**. This fragment can adopt both cyclic and open-chain configurations, with the cyclic form only ΔE = 0.65 kcal/mol less stable than the open form, suggesting coexistence of both species [46,47].

Steps 3 and 4. Alternatively, [C_3_H_6_S_2_∙⁺] can lose a thiyl radical (SH∙) to generate [C_3_H_5_OS_2_⁺] at m/z 73.01. Three isomers were identified (**d1–d3**), with cyclic structures being the most stable. Conformation **d3** is ΔE = –1.94 kcal/mol more stable than **d1**, owing to charge delocalization over sulfur within a three-membered heterocycle, a stabilization analogous to pseudoaromatic effects described for the tropylium and cyclopropenium cations. Fragment **d2** can further lose a methylene carbene (:CH_2_) (step 4), producing [C_3_H_5_OS_2_⁺] (m/z 58.99) with ΔE = 109.14 kcal/mol. Although not the primary route, this process cannot be excluded under the high-energy conditions of EI [46–48].

Step 5. The loss of H₂S from the BAL radical cation produces the [C_3_H_6_OS·⁺] ion with m/z 90.01. Three isomeric structures (**e1, e2** and **e3**) were located; e1 and e2 have very similar enthalpies (**e2** is only 3.15 kcal/mol lower in energy than **e1**), whereas **e3** is significantly more stable, lying 18.59 kcal/mol below **e1**. In all cases the species is a thiilic radical cation in which sulfur bears the formal positive charge and the unpaired electron; in e1 this charge–spin density is largely localized on S, while in **e2** the S–C–C dihedral angle of 123.54° enables additional S–C conjugation. In contrast, **e3** maximizes conjugation between S and the enolic C = C–OH π system, allowing extensive delocalization of charge and spin over S, Cα/Cβ which accounts for its markedly lower relative energy [47,49].

Step 6. Subsequent H_2_O loss from [C_3_H_6_OS∙⁺] generates [C_3_H_4_S∙⁺] at m/z 72.00. Relative energies indicates that fragmentation from **e2** is energetically more favorable (ΔE = 3.15 kcal/mol relative to **e1**), suggesting both conformers can coexist under experimental conditions.

Step 7. For [C_3_H_4_S∙⁺], two isomers were proposed: an open-chain (f1) and a cyclic (f2) form. The cyclic structure is more stable due to pseudoaromatic stabilization and charge delocalization within the ring. Further fragmentation of [C_3_H_4_S∙⁺] produces [C_2_HS⁺] at m/z 56.98 and a methyl radical (∙CH_3_), with a very high ΔE of 119.79 kcal/mol, which may contribute to its lower experimental abundance in the spectrum [47–49]. While relative energetics can suggest qualitative trends, peak intensities under EI conditions are also influenced by kinetic and statistical factors beyond simple ΔE differences.

Step 8. Finally, [C_3_H_6_OS∙⁺] can lose a thiyl radical (SH∙) to generate [C_3_H_5_O⁺] at m/z 57.03, the second most abundant ion in the spectrum. The proposed cationic structure contains conjugated C = C bonds, where charge delocalization is further stabilized by inductive effects and the participation of oxygen lone pairs.

BAL was analyzed experimentally using a Shimadzu LCMS-8050 triple quadrupole mass spectrometer equipped with an electrospray ionization (ESI) source. Spectra were acquired in positive mode at –40 V, –20 V, and –10 V [31–34] to compare with simulated spectra reported in DrugBank. The spectrum at –40 V showed correspondence only with the m/z 61 signal, while that at –20 V again matched solely at m/z 61. At –10 V, partial agreement was observed, with a single common peak at m/z 107 (see S1–S3 Figs in the Supporting Information).

### 3.2. Molecular Conformations and Harmonic Vibrational Frequencies

**Fig 3:**
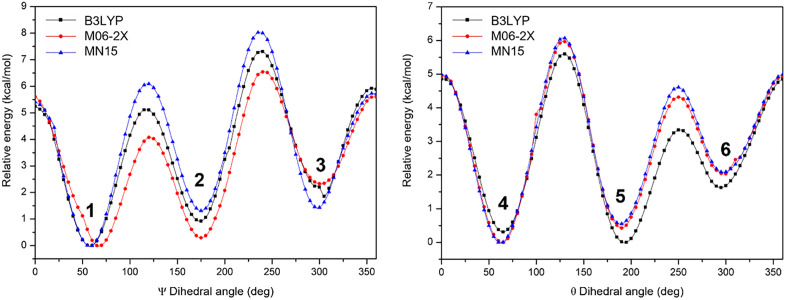
Potential energy profiles for the rotation of the dihedral angles ψ and θ for BAL calculated with the B3LYP, M06-2X and MN15 hybrid functionals combined with the 6-31 + G(*d,p*) basis set.

The rotational energy profiles of the dihedral angles ψ = O1C1C2S1 and θ = S1C2C3S2 are presented in Fig 3. Both dihedral angles show six local minima, which we will denote as BAL-1 to BAL-6. However, BAL-6 corresponds to the BAL-1 conformation upon completion of the rotation, so we will only discuss these five conformations. Conformers BAL-1, BAL-2, and BAL-3 are related to the ψ rotation, and conformers BAL-4 and BAL-5 are related to the θ rotation. No additional minima were detected when the grid spacing was reduced around the low-energy regions.

**Fig 4:**
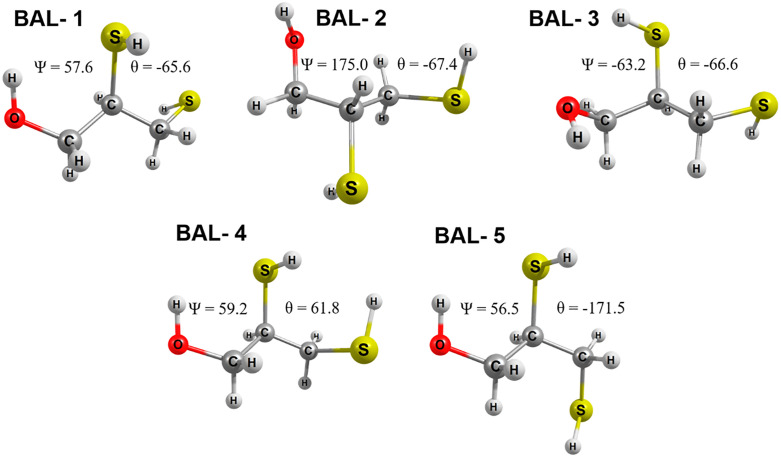
BAL isomers at the B3LYP/6-311++G(*3df,3pd*), M06-2X/6-311++G(*3df,3pd*) and MN15/6-311++G(*3df,3pd*).Values of the dihedral angles are at MN15/6-311++G(*3df,3pd*).

Fig 4 shows the equilibrium geometries of the conformers BAL-1 to BAL-5, reoptimized with the base set 6−311++G(*3df,3pd*). Conformational analysis of the dihedral angle θ yields the conformers BAL-4 and BAL-5, which correspond to the most energetically stable structures. BAL-4 exhibits a gauche (±60°) arrangement, whereas BAL-5 adopts an anti (180°) arrangement with respect to the relative orientation of the thiol (–SH) groups on C2 and C3. These geometries minimize dipole–dipole repulsion, which is particularly important in a three-carbon framework bearing three polar functionalities (–OH and two –SH groups).

**Table 2:** Estimated average values for geometric parameters for BAL calculated at the B3LYP/6-311++G(*3df,3pd*), M06-2X/6-311++G(*3df,3pd*) and MN15/6-311++G(*3df,3pd*) levels of theories. Bond length units are in angstroms (Å) and angles in degrees.

	BAL-1	BAL-2	BAL-3	BAL-4	BAL-5
*r*H_1_-O_1_	0.966	0.961	0.960	0.965	0.965
*r*O_1_-C_1_	1.403	1.416	1.416	1.406	1.403
*r*C_1_-C_2_	1.529	1.530	1.529	1.527	1.526
*r*C_2_-C_3_	1.525	1.526	1.529	1.527	1.524
*r*C_2_-S_1_	1.822	1.819	1.821	1.823	1.829
*r*C_3_-S_2_	1.813	1.815	1.813	1.818	1.820
*r*S-H^*a*^	1.334	1.339	1.339	1.339	1.339
*r*C-H^*a*^	1.092	1.091	1.091	1.091	1.091
∠C_1_-C_2_-C_3_	111.0	111.0	111.0	113.0	113.0
*dihedral* (ψ)	57.6	174.4	−62.8	59.1	56.8
*dihedral* (θ)	−65.4	−67.7	−66.1	63.3	−169.3

^*a*^Average values for S_1_-H and S_2_-H; and average values for C_1_-H, C_2_-H and C_3_-H bond lengths (see Fig 4).

**Scheme 2:**
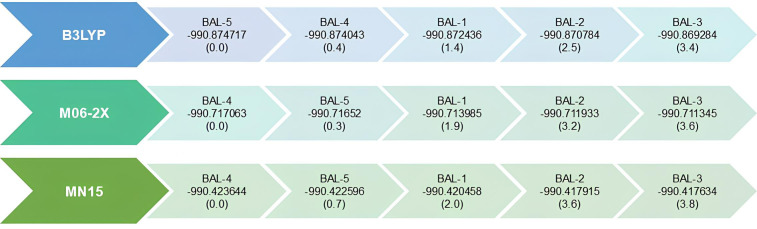
Calculated relative conformational energies for BAL isomers at the B3LYP/6-311++G(*3df,3pd*), M06-2X/6-311++G(*3df,3pd*) and MN15/6-311++G(*3df,3pd*). SCF energies values are in Hartrees (plane text) and energy differences in kcal/mol (in parentheses).

Regarding the dihedral angle ψ, the BAL-2 and BAL-3 configurations correspond to the least stable conformers. BAL-2 and BAL-3 are the anti (180°) and gauche (±60°) configurations that occur between the (-OH) group and the thiol (-SH) group located on C2, respectively. From this, we can deduce that repulsive dipole-dipole effects between the hydroxyl and thiol groups lead to less stable structures with energy differences > 2.0 kcal/mol, as reported in Scheme 2.

The calculated relative energies (Scheme 2) show that the most stable conformer depends on the functional employed. At the B3LYP/6–311++G(*3df,3pd*) level, BAL-5 is predicted as the lowest-energy conformer, following the order BAL-5 < BAL-4 < BAL-1 < BAL-2 < BAL-3. In contrast, both M06-2X and MN15 functionals identify BAL-4 as the most stable structure, with the trend BAL-4 < BAL-5 < BAL-1 < BAL-2 < BAL-3. The structural parameters of all isomers, as calculated with the B3LYP, M06-2X, and MN15 functionals, show no significant differences. Average values for bond lengths, bond angles, and dihedral angles ψ and θ for each conformer are reported in Table 1. Complete data for all calculated parameters are provided in the Supporting Information (S1 Table).

The structural parameters calculated with the B3LYP, M06-2X, and MN15 functionals combined with the 6–311++G(*3df,3pd*) basis set show average bond distances for C-H, H-O, C-O, C-C, C-S, and S-H of 1.090 Å, 0.965 Å, 1.404 Å, 1.527 Å, 1.825 Å, and 1.342 Å, respectively, across all conformers (see Table 2). These are typical values for organic compounds [34–36].

The vibrational frequencies calculated with the B3LYP, M06-2X, and MN15 functionals combined with the 6–311++G(*3df,3pd*) base set, were compared with experimental data and theoretical calculations (B3LYP/6-31G(*d,p*)) reported by Rajalakshmi and Kalaiarasi [24]. These results are presented in the supporting information in S2 Table.

The frequencies reported in S2 Table showing the characteristic signal for the hydroxyl (OH) group with a calculated value of 3840 cm ^−1^, corresponding to the experimental value of 3428 cm ^−1^. The vibrational stretching frequencies corresponding to the thiol (SH) groups were identified with calculated values at 2675 cm ^−1^ and 2671 cm ^−1^, and experimental values at 2873 cm ^−1^ and 2546 cm ^−1^. Likewise, the signal corresponding to the C-O stretching was identified with a calculated frequency at 1026 cm ^−1^ compared to the experimental 1040 cm ^−1^. Finally, the signals corresponding to a C-S-H scissoring vibration, experimentally reported at 986 and 783 cm ^−1^, correspond to the calculated frequencies at 921 and 796 cm ^−1^.

These results indicate that the use of more extensive basis sets does not provide significant improvements in the accuracy of harmonic vibrational frequencies. This observation aligns with what was reported by Mitra and Roy in 2020, in their study of vibrational frequency convergence for organic compounds functionalized with carbonyl, amino, and fluorine groups [50]. The authors demonstrated that the 6-31G(*d,p*) basis set offers an optimal balance between accuracy and computational cost for this type of calculation in medium-sized organic compounds. Similarly, Leo Radom and collaborators report poor performance of extended Pople-type basis sets, as they exhibit small contractions for HF, but when used with MP2 calculations, they show contractions that affect valence-orbital-hole configurations and, therefore, the calculation of valence correlation, negatively impacting the performance of the primitive set [51]. See S3 and S4 Tables for atomic coordinates and energies for BAL from SCF and vibrational calculations, respectively.

### 3.3. Computational thermochemistry

#### 3.3.1. Enthalpy of formation from composite methods.

The enthalpy of formation was estimated from the equilibrium structure BAL-3 using the total atomization energy (*ΣD*_*0*_) calculated with the G3MP3B3, G3B3, G4MP2, and G4 composite methods. The enthalpies of formation at 0 K (*ΔH°*_*f,0K*_) were calculated from the 0K enthalpies of formation of H, C, S, and O atoms, which are 51.63 ± 0.0, 169.98 ± 0.1, 65.66 ± 0.1, and 58.99 ± 0.0 kcal/mol, respectively [41]. After obtaining the *ΔH°*_*f,0K*_ values, they were transformed to a temperature of 298 K using the calculated thermal corrections and ZPE, and the sum of the (*H°*_*298K*_*–H°*_*0K*_) contributions for H, C, S, and O of 1.01, 0.25, 1.05, and 1.04 kcal/mol, respectively [52](see Table 3).

**Table 3:** Thermochemical data for BAL calculated from atomization energies in kcal/mol.

Level of theory	BAL-3 (values in kcal/mol)			
	Σ*D*_*0*_	*∆H°*_*f,0K*_	*∆H°*_*f,298K*_	*∆G°*_*f,298K*_
G4	1151.6	−38.3	−44.4	−18.0
G4MP2	1153.4	−40.1	−46.2	−19.8
BAC-G3B3		−37.3	−43.3	
BAC-G3MP2B3		−36.8	−42.8	
G3B3//B3LYP/ 6-311++G(*3df,3pd*)	1152.0	−38.7	−45.7	−21.0
G3MP2B3//B3LYP/ 6-311++G(*3df,3pd*)	1153.4	−40.1	−47.1	−22.3
G3B3	1151.4	−38.1	−44.1	−17.5
G3MP2B3	1153.3	−40.0	−46.0	−19.4
Mean ± Std. Dev.	1152.5 ± 1.0	−39.2 ± 1.0	−45.6 ± 1.1	−19.7 ± 1.8

It can be observed that the atomization energy values ΣD_0_ calculated using the G*n* methods have a standard deviation of 1.0 kcal/mol. However, there is a larger difference of approximately 2.0 kcal/mol between the values obtained by the G3 and G4 versions (G4-G4MP2 and G3B3-G3MP2B3) for the ΔH°_f,0K_, ΔH°_f,298K_, and ΔG°_f,298K_ values estimated by these G*n* methods, even when employing the B3LYP/6–311++G(*3df,3pd*) modification. Bond additivity corrections (BACs) were used to improve the errors associated with the electronic energy of the BAL; the resulting values are presented in Table 4.

**Table 4:** Bond additivity parameters (in kcal/mol) for the G3MP2B3 and G3B3 composited methods.

BAC parameters	G3MP2B3	G3B3
*E*_*BAC-atom*_	−3.934	−0.737
*E*_*BAC-molecule*_	−2.860	−3.180
*ΣE*_*BAC-bond*_ (*A*_*i*_*A*_*j*_)	3.638	3.075
BAC-total	−3.156	−0.842

The Gibbs free energy values at 298 K were obtained from the equation *ΔG°*_*f,298K*_ = *ΔH°*_*f,298K*_ − T(S°_(M,298K)_−ΣS°_(X,298K)_). The entropy values at 298 K for H, C, S, and O atoms correspond to 27.42 ± 0.004, 37.787 ± 0.21, 40.112 ± 0.008, and 38.494 ± 0.005 kcal/mol, respectively [41]. The value of S°_(M, 298K)_ corresponds to the difference of *H*_*corr*_ *– G*_*corr*_ with respect to the internal energy.

The mean and standard deviation values reported in Table 3 were calculated considering only composite methods. BAC-corrected values were excluded from this statistical analysis, as they are derived from deterministic corrections applied to the same underlying electronic energies (see S5 Table).

The obtained values are presented in Table 3, and the most stable value is obtained by the G3B3 method. It is noteworthy that the implementation of the more extended 6–311++G(*3df,3pd*) basis set in the G3B3 and G3MP2B3 methods produces higher Gibbs free energy values of 21.0 and 22.3 kcal/mol, respectively.

## 4. Conclusions

In this study, the structural stability, fragmentation pathways, and thermochemical properties of dimercaprol (BAL) were investigated using a combination of high-resolution mass spectrometry and advanced quantum chemical calculations. The GC/MS-QTOF analysis revealed multiple fragmentation routes of the BAL radical cation, providing experimental evidence for the main ionic species and their energetic preferences. Theoretical calculations at the M06-2X/6–311++G(*3df,3pd*) level supported these findings, showing that the most favorable pathways involve water and hydrogen sulfide losses, as well as thiyl radical elimination, with several cyclic and resonance-stabilized structures contributing to fragmentation stability.

Conformational analysis identified five main minima (BAL-1 to BAL-5), with intramolecular hydrogen bonding and gauche/anti interactions playing critical roles in conformational stability. Among these, BAL-3 emerged as the most stable conformer across different functionals, supported by vibrational frequency comparisons with experimental FTIR and Raman data. The thermochemical study further demonstrated that enthalpy of formation values obtained with G*n* composite methods were consistent, with G3B3 providing the most stable Gibbs free energy values.

Overall, this work advances the physicochemical and thermochemical characterization of dimercaprol, bridging experimental and theoretical perspectives. The results not only clarify the fragmentation behavior of BAL under electron ionization but also provide reliable energetic and structural data that can support future pharmacological, toxicological, and environmental studies. Importantly, these insights may serve as a foundation for the rational design of BAL derivatives or analogues with improved safety and therapeutic potential.

## Supporting information

S1 Table Energies calculated with theoretical level M06-2X/6–311++G(*3df,3pd*) for dimercaprol (BAL) and its fragments correspond to those reported in Scheme 1 of the article. (DOCX)

S2 Table Results of the harmonic frequencies estimated with the B3LYP, M06-2X and MN15 functionals combined with 6−311++G(*3df,3pd*) basis set for BAL-3. (DOCX)

S3 Table Atomic coordinates (in Angstroms) calculated with an M06-2X/6–311++G(*3df,3pd*) level of theory and electron energies (E in Hartrees) of the fragments experimentally detected by mass spectrometry. (DOCX)

S4 Table Atomic coordinates (in Angstroms) calculated with DFT theory and electron energies (E in Hartrees) for BAL because of vibrational calculations. (DOCX)

S5 Table Energy data corrected by the BAC method. (DOCX)

S1 Fig Experimental mass spectrum at -10V for dimercaprol. (PNG)

S2 Fig Experimental mass spectrum at -20V for dimercaprol. (PNG)

S3 Fig Experimental mass spectrum at -40V for dimercaprol. (PNG)

## Abbreviations

BAL: British Anti-Lewisite (2,3-Dimercaptopropan-1-ol)
BAC: Bond Additivity Corrections
DMSA: Dimercaptosuccinic Acid
DMPS: Dimercaptopropane Sulfonic Acid
DFT: Density Functional Theory
FTIR: Fourier Transform Infrared Spectroscopy
GC/MS-QTOF: Gas Chromatography–Mass Spectrometry Quadrupole Time-of-Flight
GCL: Glutamate–Cysteine Ligase
GSH: Glutathione
HPC: High-Performance Computing
MD: Molecular Dynamics
MM: Molecular Mechanics
MS/MS: Tandem Mass Spectrometry
NMR: Nuclear Magnetic Resonance
PM6: Parametric Method 6 (semi-empirical calculation)
SCF: Self-Consistent Field
ZPE: Zero-Point Energy
