# Supplementary material for: Dimercaprol (BAL): Insights into conformational stability, fragmentation pathways via tandem LR-ESI, HR-EI mass spectrometry, and gas-phase thermochemical properties from quantum chemical calculations
Source: PLoS One. 2026 Jun 1;21(6):e0349950. doi: 10.1371/journal.pone.0349950 (PMC13225642; doi:10.1371/journal.pone.0349950)
Supplement: S1 Table — (DOCX) [file pone.0349950.s001.docx]

**Table S1.** Energies calculated with theoretical level M06-2X/6-311++G(*3df,3pd*) for dimercaprol (BAL) and its fragments correspond to those reported in scheme 1 of the article.

| Steps | Reaction | Reactant | Product 1 | Product 2 | Reactant energy   (Hartrees) | Product 1 Energy  (Hartrees) | Product 2 Energy  (Hartrees) | Delta E  (Hartrees) | Delta E (kcal/mol) |
| --- | --- | --- | --- | --- | --- | --- | --- | --- | --- |
| 1 | E1 | a = Dimercaprol | b1 = m/z 106 | H_2_O | -990.711346 | -913.946510 | -76.427012 | 0.337824 | 211.98587 |
| 1 | E2 | a = Dimercaprol | b2 = m/z 106 | H_2_O | -990.711346 | -913.967405 | -76.427012 | 0.316929 | 198.87366 |
| 2 | E3 | b1 = m/z 106 | c1 = m/z 59 aliphatic | CH_2_SH* | -913.946510 | -475.826887 | -438.024626 | 0.094997 | 59.61094 |
| 2 | E4 | b2 = m/z 106-3 | c1 = m/z 59 aliphatic | CH_2_SH* | -913.967405 | -475.826887 | -438.024626 | 0.115893 | 72.72315 |
| 2 | E5 | b1 = m/z 106 | c2 = m/z 59 cyclic | CH_2_SH* | -913.946510 | -475.825856 | -438.024626 | 0.096028 | 60.25799 |
| 2 | E6 | b2 = m/z 106-3 | c2 = m/z 59 cyclic | CH_2_SH* | -913.967405 | -475.825856 | -438.024626 | 0.116924 | 73.37020 |
| 3 | E7 | b1 = m/z 106 | d1 = m/z73 | SH* | -913.946510 | -515.146297 | -398.734874 | 0.065339 | 41.00013 |
| 3 | E8 | b2 = m/z 106-3 | d1 = m/z73 | SH* | -913.967405 | -515.146297 | -398.734874 | 0.086234 | 54.11234 |
| 3 | E9 | b1 = m/z 106 | d2 = m/z73 | SH* | -913.946510 | -515.123890 | -398.734874 | 0.087746 | 55.06081 |
| 3 | E10 | b2 = m/z 106-3 | d2 = m/z73 | SH* | -913.967405 | -515.123890 | -398.734874 | 0.108642 | 68.17302 |
| 3 | E11 | b1 = m/z 106 | d3 = m/z73 | SH* | -913.946510 | -515.149393 | -398.734874 | 0.062243 | 39.05782 |
| 3 | E12 | b2 = m/z 106-3 | d3 = m/z73 | SH* | -913.967405 | -515.149393 | -398.734874 | 0.083139 | 52.17003 |
| 4 | E13 | d1 = m/z73 | c1 = m/z 59 aliphatic | CH_2_** | -515.146297 | -475.826887 | -39.123081 | 0.196330 | 123.19761 |
| 4 | E14 | d2 = m/z73 | c1 = m/z 59 aliphatic | CH_2_** | -515.123890 | -475.826887 | -39.123081 | 0.173923 | 109.13693 |
| 4 | E15 | d3 = m/z73 | c1 = m/z 59 aliphatic | CH_2_** | -515.149393 | -475.826887 | -39.123081 | 0.199425 | 125.13992 |
| 4 | E16 | d1 = m/z73 | c2 = m/z 59 cyclic | CH_2_** | -515.146297 | -475.825856 | -39.123081 | 0.197361 | 123.84466 |
| 4 | E17 | d2 = m/z73 | c2 = m/z 59 cyclic | CH_2_** | -515.123890 | -475.825856 | -39.123081 | 0.174954 | 109.78398 |
| 4 | E18 | d3 = m/z73 | c2 = m/z 59 cyclic | CH_2_** | -515.149393 | -475.825856 | -39.123081 | 0.200456 | 125.78697 |
| 5 | E19 | a = Dimercaprol | e1 = m/z 90 | H_2_S | -990.711346 | -590.989517 | -399.383959 | 0.337870 | 212.01428 |
| 5 | E20 | a = Dimercaprol | e2 = m/z 90 | H_2_S | -990.711346 | -590.984489 | -399.383959 | 0.342898 | 215.16939 |
| 5 | E21 | a = Dimercaprol | e3 = m/z 90 | H_2_S | -990.711346 | -591.019149 | -399.383959 | 0.308238 | 193.42027 |
| 6 | E22 | e1 = m/z 90 | f1 = m/z 72 | H_2_O | -590.989517 | -514.528534 | -76.427012 | 0.033971 | 21.31691 |
| 6 | E23 | e2 = m/z 90 | f1 = m/z 72 | H_2_O | -590.984489 | -514.528534 | -76.427012 | 0.028943 | 18.16181 |
| 6 | E24 | e3 = m/z 90 | f1 = m/z 72 | H_2_O | -591.019149 | -514.528534 | -76.427012 | 0.063603 | 39.91107 |
| 6 | E25 | e1 = m/z 90 | f2 = m/z 72 cyclic | H_2_O | -590.989517 | -514.529676 | -76.427012 | 0.032830 | 20.60068 |
| 6 | E26 | e2 = m/z 90 | f2 = m/z 72 cyclic | H_2_O | -590.984489 | -514.529676 | -76.427012 | 0.027802 | 17.44557 |
|  | E27 | e3 = m/z 90 | f2 = m/z 72 cyclic | H_2_O | -591.019149 | -514.529676 | -76.427012 | 0.062461 | 39.19446 |
| 7 | E28 | f1 = m/z 72 | g = m/z 57 thiol | CH_3_* | -514.528534 | -474.513693 | -39.823935 | 0.190906 | 119.79433 |
| 7 | E29 | f2 = m/z 72 cyclic | g = m/z 57 thiol | CH_3_* | -514.529676 | -474.513693 | -39.823935 | 0.192048 | 120.51056 |
| 8 | E30 | e1 = m/z 90 | h = m/z 57 ol | SH* | -590.989517 | -192.211327 | -398.734874 | 0.043317 | 27.18162 |
| 8 | E31 | e2 = m/z 90 | h = m/z 57 ol | SH* | -590.984489 | -192.211327 | -398.734874 | 0.038289 | 24.02651 |
| 8 | E32 | e3 = m/z 90 | h = m/z 57 ol | SH* | -591.019149 | -192.211327 | -398.734874 | 0.072948 | 45.77509 |
