# Supplementary material for: Dimercaprol (BAL): Insights into conformational stability, fragmentation pathways via tandem LR-ESI, HR-EI mass spectrometry, and gas-phase thermochemical properties from quantum chemical calculations
Source: PLoS One. 2026 Jun 1;21(6):e0349950. doi: 10.1371/journal.pone.0349950 (PMC13225642; doi:10.1371/journal.pone.0349950)
Supplement: S2 Table — (DOCX) [file pone.0349950.s002.docx]

**Table S2.** Results of the harmonic frequencies estimated with the B3LYP, M06-2X and MN15 functionals combined with 6-311++G(3df,3pd) basis set for BAL-3.

| Vibrational Assignments | Exp.  Frequencies | B3LYP  6-31G(d,p) | Harmonic Frequencies | | |  |
| --- | --- | --- | --- | --- | --- | --- |
|  |  |  | **B3LYP** | **M06-2X** | **MN15** | **Averag.** |
|  |  |  | **6-311++G(*3df,3pd*)** | | |  |
| O-H stretching | 3428 | 3821 | 3840 | 3904 | 3913 | 3886±40 |
| CH_2_ asymmetric stretching |  | 3120 | 3103 | 3133 | 3161 | 3132±29 |
| CH_2_ asymmetric stretching |  | 3118 | 3103 | 3127 | 3144 | 3125±21 |
| CH stretching |  | 3076 | 3060 | 3091 | 3124 | 3092±32 |
| CH_2_ symmetric stretching |  | 3048 | 3039 | 3065 | 3077 | 3060±19 |
| CH_2_ asymmetric stretching |  | 2996 | 2994 | 3031 | 3049 | 3025±28 |
| SH stretching | 2873 | 2671 | 2675 | 2733 | 2744 | 2717±37 |
| SH stretching | 2546 | 2669 | 2671 | 2722 | 2740 | 2711±36 |
| CH_2_ scissoring |  | 1508 | 1493 | 1506 | 1479 | 1493±14 |
| CH_2_ scissoring |  | 1478 | 1464 | 1468 | 1445 | 1459±12 |
| CH_2_ wagging, OH bending | 1419 | 1417 | 1408 | 1414 | 1392 | 1405±11 |
| CH_2_ twisting, OH bending, CH bending |  | 1394 | 1381 | 1389 | 1361 | 1377±14 |
| CH_2_ twisting, C-H twisting | 1313 | 1351 | 1338 | 1345 | 1327 | 1337±9 |
| CH_2_ twisting, C-H bending, O-H bending |  | 1298 | 1290 | 1298 | 1284 | 1291±7 |
| CH_2_ wagging, C-H bending | 1272 | 1261 | 1254 | 1262 | 1244 | 1253±9 |
| CH_2_ twisting, O-H bending, C-H bending | 1237 | 1223 | 1217 | 1226 | 1205 | 1216±11 |
| CH_2_ twisting, O-H bending, S-H bending | 1170 | 1210 | 1204 | 1210 | 1187 | 1200±12 |
| O-C-C asymmetric stretching, C-H bending, S-H bending |  | 1102 | 1088 | 1135 | 1132 | 1118±26 |
| S-H rocking, O-H bending, C-H bending |  | 1072 | 1064 | 1090 | 1079 | 1077±13 |
| C-O stretching, O-H bending,  C-H bending, S-H bending | 1040 | 1033 | 1026 | 1042 | 1031 | 1033±8 |
| S-H scissoring, C-H twisting, O-H bending |  | 951 | 942 | 964 | 954 | 953±11 |
| C-S-H scissoring, CH bending | 986 | 925 | 921 | 934 | 928 | 927±7 |
| S-H bending, C-H_2_ rocking, O-H bending |  | 876 | 870 | 896 | 890 | 886±14 |
| C-S-H scissoring, C-H bending | 783 | 801 | 796 | 816 | 811 | 808±10 |
| C-S-H rocking |  | 745 | 740 | 759 | 752 | 750±10 |
| C-S-H rocking, CH_2_ rocking |  | 640 | 635 | 661 | 666 | 654±17 |
| C-C-O scissoring |  | 565 | 565 | 584 | 581 | 577±10 |
| S-H twisting, CH_2_ rocking |  | 383 | 375 | 390 | 383 | 383±8 |
| H-S-C wagging, SH bending, |  | 367 | 329 | 344 | 337 | 337±8 |
| S-H twisting, CH_2_ rocking |  | 324 | 289 | 313 | 300 | 301±12 |
| S-H twisting, O-H twisting |  | 271 | 253 | 281 | 260 | 265±15 |
| CH_2_ rocking, S-H bending |  | 255 | 223 | 263 | 248 | 244±20 |
| O-H twisting, SH bending |  | 183 | 179 | 253 | 181 | 204±42 |
| CH_2_ rocking, SH bending |  | 156 | 155 | 175 | 162 | 164±10 |
| CH_2_ rocking, OH bending |  | 103 | 104 | 116 | 116 | 112±7 |
| CH_2_ rocking |  | 97 | 93 | 105 | 99 | 99±6 |
