# Supplementary material for: Dimercaprol (BAL): Insights into conformational stability, fragmentation pathways via tandem LR-ESI, HR-EI mass spectrometry, and gas-phase thermochemical properties from quantum chemical calculations
Source: PLoS One. 2026 Jun 1;21(6):e0349950. doi: 10.1371/journal.pone.0349950 (PMC13225642; doi:10.1371/journal.pone.0349950)
Supplement: S3 Table — (DOCX) [file pone.0349950.s003.docx]

**Table S3.** Atomic coordinates (in Angstroms) calculated with an M06-2X/6-311++G(*3df,3pd*) level of theory and electron energies (E in Hartrees) of the fragments experimentally detected by mass spectrometry.

| **BAL (a)**  **E =** -990.40052228   \| C \| -0.258582 \| 0.186446 \| -0.203010 \| \| --- \| --- \| --- \| --- \| \| H \| 0.240715 \| 0.651294 \| -1.050790 \| \| C \| -1.772446 \| 0.260336 \| -0.351847 \| \| H \| -2.117230 \| -0.022233 \| -1.342778 \| \| H \| -2.284637 \| -0.340945 \| 0.395103 \| \| C \| 0.317436 \| -1.196537 \| 0.027155 \| \| H \| 0.089971 \| -1.814602 \| -0.847361 \| \| H \| -0.148796 \| -1.654375 \| 0.905926 \| \| S \| 0.080977 \| 1.264846 \| 1.254728 \| \| H \| 0.947608 \| 2.122795 \| 0.693316 \| \| S \| -2.160784 \| 2.024104 \| -0.055019 \| \| H \| -2.949770 \| 1.884184 \| 1.021216 \| \| O \| 1.692865 \| -0.998754 \| 0.200529 \| \| H \| 2.142374 \| -1.832316 \| 0.362341 \| | **m/z 106 b1**  **E = -913.967405**   \| C \| 0.693895 \| 0.956800 \| 0.011292 \| \| --- \| --- \| --- \| --- \| \| H \| 0.774445 \| 1.581698 \| 0.905785 \| \| H \| 0.772863 \| 1.619140 \| -0.856067 \| \| C \| -0.677013 \| 0.362076 \| 0.002141 \| \| C \| -2.021164 \| 1.369807 \| -0.009543 \| \| H \| -1.886242 \| 2.443439 \| -0.015833 \| \| H \| -3.031574 \| 0.986564 \| -0.009346 \| \| S \| 2.008579 \| -0.272509 \| -0.009792 \| \| H \| 3.006599 \| 0.616949 \| 0.064996 \| \| S \| -0.798335 \| -1.327568 \| 0.005102 \| \| H \| -2.141467 \| -1.401942 \| 0.004159 \| |
| --- | --- | --- | --- | --- | --- | --- | --- | --- | --- | --- | --- | --- | --- | --- | --- | --- | --- | --- | --- | --- | --- | --- | --- | --- | --- | --- | --- | --- | --- | --- | --- | --- | --- | --- | --- | --- | --- | --- | --- | --- | --- | --- | --- | --- | --- | --- | --- | --- | --- | --- | --- | --- | --- | --- | --- | --- | --- | --- | --- | --- | --- | --- | --- | --- | --- | --- | --- | --- | --- | --- | --- | --- | --- | --- | --- | --- | --- | --- | --- | --- | --- | --- | --- | --- | --- | --- | --- | --- | --- | --- | --- | --- | --- | --- | --- | --- | --- | --- | --- | --- | --- |
| **m/z 106 b2**  **E = -913.946508**   \| C \| -3.287617 \| 2.032911 \| 1.257405 \| \| --- \| --- \| --- \| --- \| \| H \| -2.217617 \| 2.032728 \| 1.257502 \| \| H \| -3.644112 \| 3.041777 \| 1.257307 \| \| C \| -3.801189 \| 1.307116 \| 2.514810 \| \| S \| -3.880961 \| 1.193818 \| -0.195959 \| \| H \| -3.446560 \| 1.812301 \| -1.265948 \| \| S \| -3.208191 \| -0.371203 \| 2.514941 \| \| C \| -3.287803 \| 2.033042 \| 3.772214 \| \| H \| -3.644268 \| 3.041920 \| 3.772134 \| \| H \| -3.644636 \| 1.528757 \| 4.645865 \| \| H \| -2.217803 \| 2.032828 \| 3.772294 \| \| C \| -3.287617 \| 2.032911 \| 1.257405 \| | **m/z 59 c1**  **E = -475.826887**   \| C \| 1.083351 \| -0.461147 \| 0.000058 \| \| --- \| --- \| --- \| --- \| \| H \| 1.475905 \| -0.864350 \| 0.927012 \| \| H \| 1.475747 \| -0.865458 \| -0.926480 \| \| C \| 0.458529 \| 0.840021 \| -0.000494 \| \| H \| 0.668296 \| 1.902692 \| 0.001168 \| \| S \| -0.804452 \| -0.152883 \| 0.000057 \| \|  \|  \|  \|  \| \|  \|  \|  \|  \| \|  \|  \|  \|  \| |
| **m/z 59 c2**  **E = -475.825856**   \| C \| 1.821586 \| -0.139250 \| 0.019262 \| \| --- \| --- \| --- \| --- \| \| H \| 2.431699 \| -1.012186 \| 0.122437 \| \| H \| 2.276905 \| 0.826542 \| -0.050239 \| \| C \| 0.426170 \| -0.260865 \| -0.024843 \| \| S \| -1.186713 \| -0.401437 \| -0.075822 \| \| H \| -1.744155 \| -1.583861 \| 0.009223 \| | **m/z 73 d1**  **E = -515.146297**   \| C \| 0.238084 \| -0.065167 \| -0.135966 \| \| --- \| --- \| --- \| --- \| \| C \| 1.683973 \| -0.613987 \| 0.166152 \| \| H \| 1.951800 \| -0.834821 \| 1.178280 \| \| C \| 1.516415 \| 0.797570 \| -0.350812 \| \| H \| 1.711925 \| 1.666314 \| 0.242451 \| \| S \| -1.532864 \| -0.239695 \| -0.094955 \| \| H \| -2.090829 \| 0.871738 \| -0.506649 \| \| H \| 1.846611 \| 1.045711 \| -1.337877 \| \| H \| 2.079654 \| -1.371616 \| -0.477537 \| |
| **m/z 73 d2**  **E = -515.123890**   \| C \| 0.426554 \| 0.007461 \| -0.001904 \| \| --- \| --- \| --- \| --- \| \| C \| 0.434247 \| -1.381873 \| 0.001693 \| \| H \| 1.370679 \| -1.924648 \| -0.001347 \| \| H \| -0.479553 \| -1.966396 \| 0.008936 \| \| C \| 0.478260 \| 1.394887 \| 0.008011 \| \| H \| 1.434066 \| 1.903570 \| 0.007864 \| \| H \| -0.410793 \| 2.014315 \| 0.017268 \| \| S \| -1.325415 \| -0.098131 \| 0.011178 \| \| H \| -1.560155 \| 1.224751 \| 0.008211 \| | **m/z 73 d3**  **E = -515.149393**   \| C \| -1.209793 \| 0.170069 \| -0.285043 \| \| --- \| --- \| --- \| --- \| \| C \| -0.475401 \| -1.098559 \| 0.187030 \| \| H \| 0.386304 \| -1.015180 \| 0.815849 \| \| H \| -0.826845 \| -2.063387 \| -0.113791 \| \| C \| -2.221380 \| 0.683020 \| 0.456671 \| \| H \| -2.509822 \| 0.206571 \| 1.370289 \| \| H \| -2.731640 \| 1.564469 \| 0.128673 \| \| S \| -0.729957 \| 0.962663 \| -1.804895 \| \| H \| 0.196227 \| 1.855652 \| -1.558208 \| |
| **m/z 90 e1**  **E = -590.989517**   \| C \| 0.339873 \| 0.488316 \| -0.000014 \| \| --- \| --- \| --- \| --- \| \| C \| 1.283749 \| 1.495230 \| -0.000035 \| \| H \| 2.165320 \| 1.157708 \| -0.503832 \| \| H \| 0.890531 \| 2.352689 \| -0.505054 \| \| C \| -1.126846 \| 0.794088 \| 0.000104 \| \| H \| -1.349583 \| 1.409073 \| -0.883651 \| \| H \| -1.349536 \| 1.408713 \| 0.884118 \| \| S \| 0.729082 \| -1.167467 \| 0.000022 \| \| O \| -1.819857 \| -0.417413 \| -0.000130 \| \| H \| -2.774317 \| -0.285042 \| 0.000129 \| \| H \| 1.527169 \| 1.755939 \| 1.008765 \| | **m/z 90 e2**  **E = -590.984480**   \| C \| 0.339873 \| 0.488316 \| -0.000014 \| \| --- \| --- \| --- \| --- \| \| C \| 1.283749 \| 1.495230 \| -0.000035 \| \| H \| 2.345774 \| 1.295147 \| 0.000082 \| \| H \| 0.970083 \| 2.531726 \| -0.000255 \| \| C \| -1.126846 \| 0.794088 \| 0.000104 \| \| H \| -1.349583 \| 1.409073 \| -0.883651 \| \| H \| -1.349536 \| 1.408713 \| 0.884118 \| \| S \| 0.729082 \| -1.167467 \| 0.000022 \| \| H \| 2.070458 \| -1.006648 \| -0.000068 \| \| O \| -1.819857 \| -0.417413 \| -0.000130 \| \| H \| -2.774317 \| -0.285042 \| 0.000129 \| |
| **m/z 72 f1**  **E = -514.528534**   \| C \| 1.858829 \| -0.347129 \| 0.101053 \| \| --- \| --- \| --- \| --- \| \| H \| 2.488574 \| -0.309918 \| -0.763203 \| \| H \| 2.289429 \| -0.297325 \| 1.079318 \| \| C \| 0.515858 \| -0.457334 \| -0.043346 \| \| H \| 0.085259 \| -0.507135 \| -1.021612 \| \| C \| -0.390503 \| -0.510890 \| 1.200535 \| \| H \| 0.040097 \| -0.461088 \| 2.178800 \| \| S \| -1.942768 \| -0.638272 \| 1.033632 \| | **m/z 72 f2**  **E = -514.529676**   \| C \| -1.245054 \| -0.018558 \| -0.492958 \| \| --- \| --- \| --- \| --- \| \| C \| -0.334550 \| -0.954304 \| 0.323719 \| \| H \| -0.651471 \| -0.957717 \| 1.345702 \| \| H \| -0.396001 \| -1.946510 \| -0.072074 \| \| C \| -2.067316 \| 1.137205 \| -0.483431 \| \| H \| -2.453560 \| 1.953146 \| 0.090987 \| \| S \| -2.000618 \| 0.327507 \| -2.092940 \| \| H \| 0.676443 \| -0.608848 \| 0.264960 \| |
| **m/z 57 thiol g**  **E = -474.513693**   \| C \| 1.837456 \| 0.152580 \| 0.128487 \| \| --- \| --- \| --- \| --- \| \| H \| 2.884200 \| 0.305374 \| 0.289359 \| \| C \| 0.466514 \| -0.047536 \| -0.082211 \| \| S \| -1.118081 \| -0.278841 \| -0.325743 \| | **m/z 57 ol h**  **E = -192.211327**   \| C \| -3.896883 \| 0.563549 \| 0.000000 \| \| --- \| --- \| --- \| --- \| \| H \| -3.540228 \| -0.445261 \| 0.000000 \| \| H \| -3.540210 \| 1.067947 \| -0.873652 \| \| C \| -3.383540 \| 1.289505 \| 1.257405 \| \| H \| -2.313540 \| 1.289323 \| 1.257502 \| \| C \| -3.896627 \| 2.741519 \| 1.257265 \| \| H \| -3.538786 \| 3.246105 \| 0.384199 \| \| O \| -3.420849 \| 3.414900 \| 2.425636 \| \| H \| -3.740130 \| 4.320250 \| 2.426074 \| |
| **SH**  **E = -398.734874**   \| S \| -1.211031 \| 0.995119 \| 0.013027 \| \| --- \| --- \| --- \| --- \| \| H \| -1.648318 \| 2.229979 \| 0.013027 \| \|  \|  \|  \|  \| | **CH_3_ rad**  **E = -39.823935**   \| C \| -1.594724 \| 0.539568 \| 0.000000 \| \| --- \| --- \| --- \| --- \| \| H \| -1.238070 \| -0.469242 \| 0.000000 \| \| H \| -1.238051 \| 1.043967 \| -0.873652 \| \| H \| -2.664724 \| 0.539582 \| 0.000000 \| \| C \| -1.594724 \| 0.539568 \| 0.000000 \| |
| **CH_2_SH rad**  **E =** -438.024626   \| C \| -2.505995 \| 1.378897 \| 0.000000 \| \| --- \| --- \| --- \| --- \| \| H \| -2.149341 \| 0.370087 \| 0.000000 \| \| H \| -2.149322 \| 1.883295 \| -0.873652 \| \| S \| -1.912652 \| 2.217989 \| 1.453364 \| \| H \| -2.347052 \| 3.453868 \| 1.452747 \| \| C \| -2.505995 \| 1.378897 \| 0.000000 \| | **CH_2_**  **E =** -39.123081   \| C \| -1.594724 \| 0.539568 \| 0.000000 \| \| --- \| --- \| --- \| --- \| \| H \| -1.238070 \| -0.469242 \| 0.000000 \| \| H \| -1.238051 \| 1.043967 \| -0.873652 \| |
| **H_2_O**  **E =** -76.427012   \| O \| -0.887436 \| 1.101065 \| 0.000000 \| \| --- \| --- \| --- \| --- \| \| H \| 0.070448 \| 1.138038 \| 0.000000 \| \| H \| -1.172332 \| 2.016348 \| 0.000000 \| | **SH_2_**  **E =** -399.38395921   \| S \| 0.083037 \| 0.618669 \| 0.197708 \| \| --- \| --- \| --- \| --- \| \| H \| 1.403589 \| 0.818612 \| 0.197708 \| \| H \| -0.169297 \| 1.930218 \| 0.197708 \| |
