# Supplementary material for: Dimercaprol (BAL): Insights into conformational stability, fragmentation pathways via tandem LR-ESI, HR-EI mass spectrometry, and gas-phase thermochemical properties from quantum chemical calculations
Source: PLoS One. 2026 Jun 1;21(6):e0349950. doi: 10.1371/journal.pone.0349950 (PMC13225642; doi:10.1371/journal.pone.0349950)
Supplement: S4 Table — (DOCX) [file pone.0349950.s004.docx]

**Table S4.** Atomic coordinates (in Angstroms) calculated with DFT theory and electron energies (E in Hartrees) for BAL because of vibrational calculations.

| **BAL** B3LYP/6-311++G(*3df,3pd*)  E = -990.86928414   \| S \| 0.574305 \| 1.672004 \| -0.087165 \| \| --- \| --- \| --- \| --- \| \| S \| -2.427591 \| -0.195307 \| -0.086026 \| \| O \| 2.317522 \| -0.964462 \| -0.607935 \| \| C \| 0.338712 \| -0.032139 \| 0.556880 \| \| C \| -0.718066 \| -0.825206 \| -0.220334 \| \| C \| 1.670770 \| -0.787521 \| 0.646060 \| \| H \| -0.011836 \| 0.112011 \| 1.580312 \| \| H \| -0.715178 \| -1.868637 \| 0.100118 \| \| H \| -0.498242 \| -0.805372 \| -1.286666 \| \| H \| 1.499858 \| -1.756707 \| 1.128218 \| \| H \| 2.363575 \| -0.220356 \| 1.264266 \| \| H \| 1.460280 \| 1.322124 \| -1.035058 \| \| H \| -2.667899 \| -0.655946 \| 1.152785 \| \| H \| 1.899096 \| -1.682889 \| -1.088908 \| | **BAL** M06-2X/6-311++G(*3df,3pd*)  E = -990.71134576   \| S \| 0.563421 \| 1.653151 \| -0.064943 \| \| --- \| --- \| --- \| --- \| \| S \| -2.393548 \| -0.210270 \| -0.115590 \| \| O \| 2.265047 \| -0.963766 \| -0.616083 \| \| C \| 0.334193 \| -0.035095 \| 0.573323 \| \| C \| -0.695468 \| -0.831900 \| -0.226271 \| \| C \| 1.665840 \| -0.781095 \| 0.650165 \| \| H \| -0.030644 \| 0.094903 \| 1.593235 \| \| H \| -0.685602 \| -1.878024 \| 0.084578 \| \| H \| -0.449246 \| -0.794631 \| -1.286946 \| \| H \| 1.508069 \| -1.745269 \| 1.144388 \| \| H \| 2.370038 \| -0.202913 \| 1.244115 \| \| H \| 1.427046 \| 1.291948 \| -1.022819 \| \| H \| -2.612786 \| -0.611624 \| 1.141469 \| \| H \| 1.818947 \| -1.673819 \| -1.082075 \| |
| --- | --- | --- | --- | --- | --- | --- | --- | --- | --- | --- | --- | --- | --- | --- | --- | --- | --- | --- | --- | --- | --- | --- | --- | --- | --- | --- | --- | --- | --- | --- | --- | --- | --- | --- | --- | --- | --- | --- | --- | --- | --- | --- | --- | --- | --- | --- | --- | --- | --- | --- | --- | --- | --- | --- | --- | --- | --- | --- | --- | --- | --- | --- | --- | --- | --- | --- | --- | --- | --- | --- | --- | --- | --- | --- | --- | --- | --- | --- | --- | --- | --- | --- | --- | --- | --- | --- | --- | --- | --- | --- | --- | --- | --- | --- | --- | --- | --- | --- | --- | --- | --- | --- | --- | --- | --- | --- | --- | --- | --- | --- | --- | --- | --- |
| **BAL** B3LYP/6-31G(*d,p*)  E = -990.73062627   \| S \| 0.573715 \| 1.668931 \| -0.112079 \| \| --- \| --- \| --- \| --- \| \| S \| -2.443979 \| -0.211649 \| -0.106479 \| \| O \| 2.316002 \| -0.937878 \| -0.617589 \| \| C \| 0.332622 \| -0.035589 \| 0.563745 \| \| C \| -0.719828 \| -0.841877 \| -0.213893 \| \| C \| 1.675693 \| -0.784415 \| 0.642307 \| \| H \| -0.017911 \| 0.114305 \| 1.590694 \| \| H \| -0.715865 \| -1.886259 \| 0.120023 \| \| H \| -0.487550 \| -0.834761 \| -1.282332 \| \| H \| 1.512054 \| -1.761660 \| 1.123348 \| \| H \| 2.368440 \| -0.215419 \| 1.267030 \| \| H \| 1.518282 \| 1.288097 \| -0.998049 \| \| H \| -2.676197 \| -0.628878 \| 1.155706 \| \| H \| 1.849828 \| -1.621354 \| -1.115886 \| |  |
