# Supplementary material for: Dimercaprol (BAL): Insights into conformational stability, fragmentation pathways via tandem LR-ESI, HR-EI mass spectrometry, and gas-phase thermochemical properties from quantum chemical calculations
Source: PLoS One. 2026 Jun 1;21(6):e0349950. doi: 10.1371/journal.pone.0349950 (PMC13225642; doi:10.1371/journal.pone.0349950)
Supplement: S5 Table — (DOCX) [file pone.0349950.s005.docx]

**Table S5. Energy data corrected by the BAC method.**

For bonds with carbon, the parameter AabExp(-alphaRab) is zero because the contribution Aii for this atom is zero. Therefore, EBAC-bond(AB) will depend on the contribution of neighboring bonds (the errors due to nearest neighbors) for the G3B3 and G3MP2B3 methods.

| G3MP2B3 | |
| --- | --- |
| **Bond** | **Ebac-bond** |
| (H1-O1), has as its near the bond O1-C1 | (0.3259*68.4380)½ e(-3*0.97) +  Bo-c(0.031+0.002)= **0.290** |
| (O1-C1), has as its near the bond H1-O1 + x2 C-H + C1-C2 | (0.113+0.031)+((0.002+0.113)*2)  +(0.002*2)= **0.378** |
| The bond (H-C1) has as its nearest neighbors the bond O1-C1, C-H and C1-C2 this value x 2 (x2 for H2 and H3) | 2*[(0.031+0.002)+(0.002+0.113)  +(0.002*2)]= (0.152*2)= **0.304** |
| Bond(C1-C2) has as neighbors the bond O1-C1, C-H x2 for the first carbon and C2-S1, C2-H4 and C2-C3 for the second carbon | (0.031+0.002)+((0.002+0.113)*2)+(0.002+0.064)  +(0.002+0.113)+(0.002*2)= **0.448** |
| Bond(C2-C3) has as neighbors the bond C1-C2, C2-S1 and C2-H4 for the first carbon (C2) and 2x C-H for the atoms H6 and H7 + C3-S2 the second carbon (C3) | (0.002*2)+(0.002+0.064)+(0.002+0.113)+  (2*(0.002+0.113))+(0.002+0.064)=**0.481** |
| Bond(C2-S1) has neighbors S1-H5, C2-H4 and x2 C-C for C1 and C3 | (0.064+0.113)+(0.002+0.113)+(0.002*4)= **0.300** |
| Bond(C3-S2) has neighbors S2-H8, x2 C-H for H6 and H7 and C2-C3 | (0.064+0.113)+(2*(0.002+0.113))+(0.002*2)=**0.411** |
| Bond(C2-H4) has x2 C-C neighbors for C1 and C3 and C2-S1 | (0.002*4)+(0.002+0.064)=**0.074** |
| Bond(S1-H5) has neighbors C2-S1 | (0.3259*519.0579)½ e(-3*1.352)+(0.002+0.064)=**0.291** |
| Bond(S2-H8) has C-S as neighbors | (0.3259*519.0579)½ e(-3*1.352)+(0.002+0.064)= **0.291** |
| 2* Bond(C3-H7) x2= has neighbors C-C, C-S and C-H for H6 and H7 | 2*((0.002*2)+(0.002+0.064)+(0.002+0.113))=**0.37** |
| **Ebac-atom total** | **-3.934** |
| **Ebac-molecule** | **-2.86** |
| **BAC-TOTAL** | **-3.156** |
| **Total** | **3.638** |
| **G3B3** | |
| Bond(H1-O1) has as a neighbor the bond O1-C1 | (1.1500*45.8645)½ e(-3*0.97) + Bo-c(-0.024+0.005)= **0.377** |
| Bond(O1-C1) has as a neighbor the bond H1-O1 + x2 C-H + C1-C2 | (0.060+-0.024)+((0.005+0.060)*2)+(0.005*2)= **0.176** |
| Bond(H-C1) has as neighbors the bond O1-C1, C-H and C1-C2 this value x 2 (x2 for H2 and H3) | 2*((-0.024+0.005)+(0.005+0.060)+(0.005*2))= (0.152*2)= **0.112** |
| Bond(C1-C2) has as neighbors the bond O1-C1, C-H x2 for the first carbon and C2-S1, C2-H4 and C2-C3 for the second carbon | (-0.024+0.005)+((0.005+0.060)*2)+(0.005+0.063)  +(0.005+0.060)+(0.005*2)=  **0.254** |
| Bond(C2-C3) has as neighbors the bond C1-C2, C2-S1 and C2-H4 for the first carbon (C2) and 2x C-H for the atoms H6 and H7 + C3-S2 the second carbon (C3) | (0.005*2)+(0.005+0.063)+(0.005+0.060)+  (2*(0.005+0.060))+(0.005+0.063)=**0.341** |
| Bond(C2-S1) has neighbors S1-H5, C2-H4 and x2 C-C for C1 and C3 | (0.063+0.060)+(0.005+0.060)+(0.005*4)= **0.208** |
| Bond(C3-S2) has neighbors S2-H8, x2 C-H for H6 and H7 and C2-C3 | (0.063+0.060)+(2*(0.005+0.060))+(0.005*2)= **0.263** |
| Bond(C2-H4) has x2 C-C neighbors for C1 and C3 and C2-S1 | (0.005*4)+(0.005+0.063)=**0.088** |
| Bond(S1-H5) has neighbors C2-S1 | (1.1500*503.9800)½ e(-3*1.352)+(0.005+0.063)= **0.485** |
| Bond(S2-H8) has C-S as neighbors | (1.1500*503.9800)½ e(-3*1.352)+(0.005+0.063)=**0.485** |
| 2* Bond(C3-H7) x2= has neighbors C-C, C-S and C-H for H6 and H7 | 2*((0.005*2)+(0.005+0.063)+(0.005+0.060))= **0.286** |
| **Ebac-atom total** | **-0.737** |
| **Ebac-molecule** | **-3.18** |
| **BAC-TOTAL** | **-0.842** |
| **TOTAL** | **3.075** |
