# Supplementary figures and images for: Dimercaprol (BAL): Insights into conformational stability, fragmentation pathways via tandem LR-ESI, HR-EI mass spectrometry, and gas-phase thermochemical properties from quantum chemical calculations
Source: PLoS One. 2026 Jun 1;21(6):e0349950. doi: 10.1371/journal.pone.0349950 (PMC13225642; doi:10.1371/journal.pone.0349950)

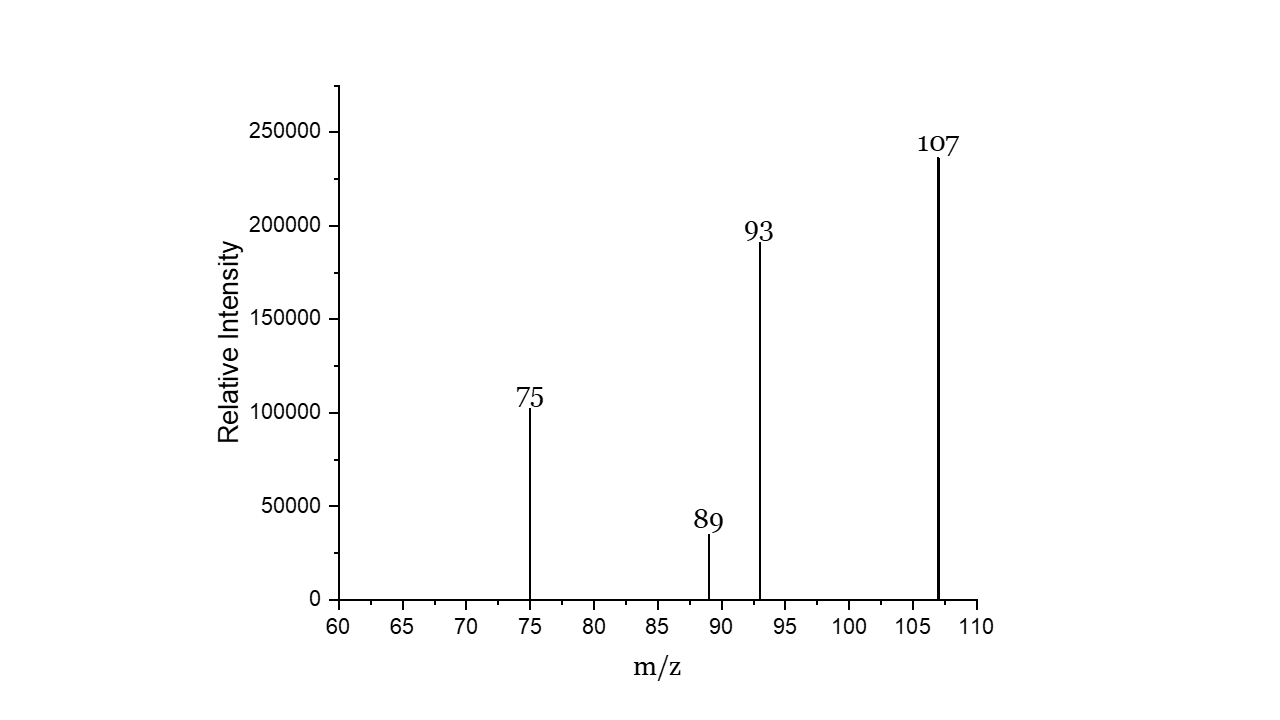

Supplement: S1 Fig — (PNG) [file pone.0349950.s006.png]

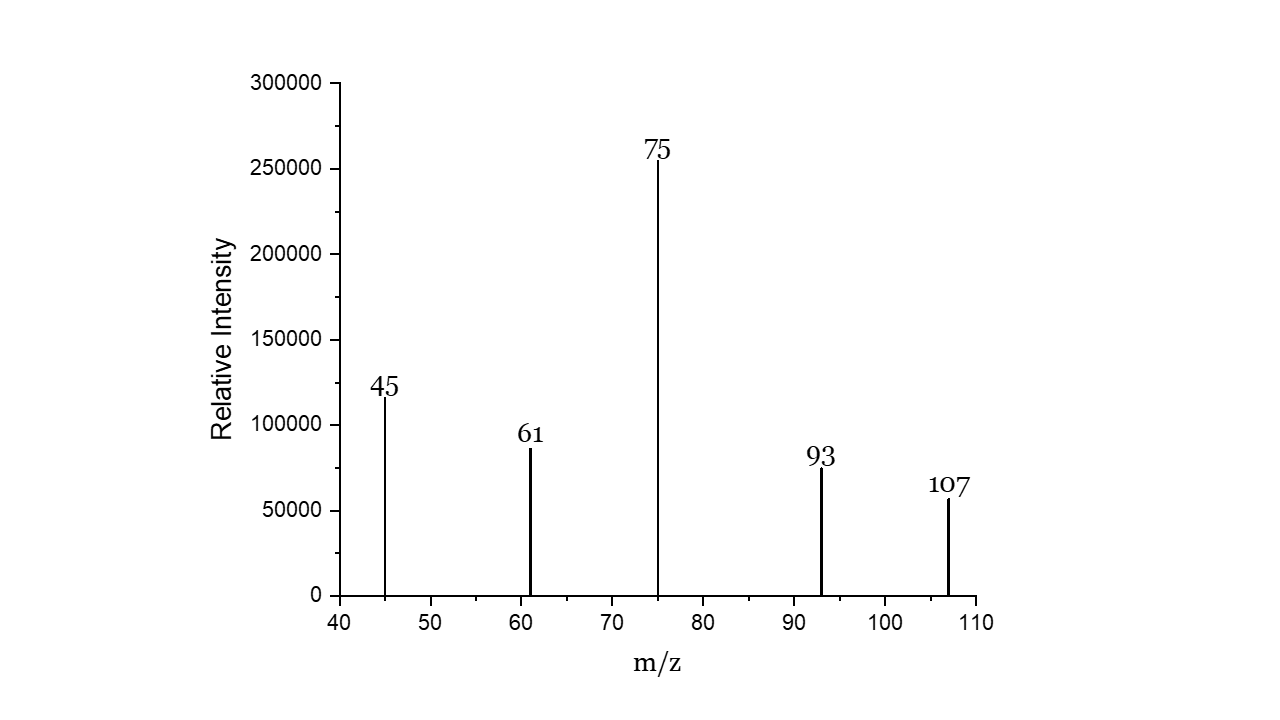

Supplement: S2 Fig — (PNG) [file pone.0349950.s007.png]

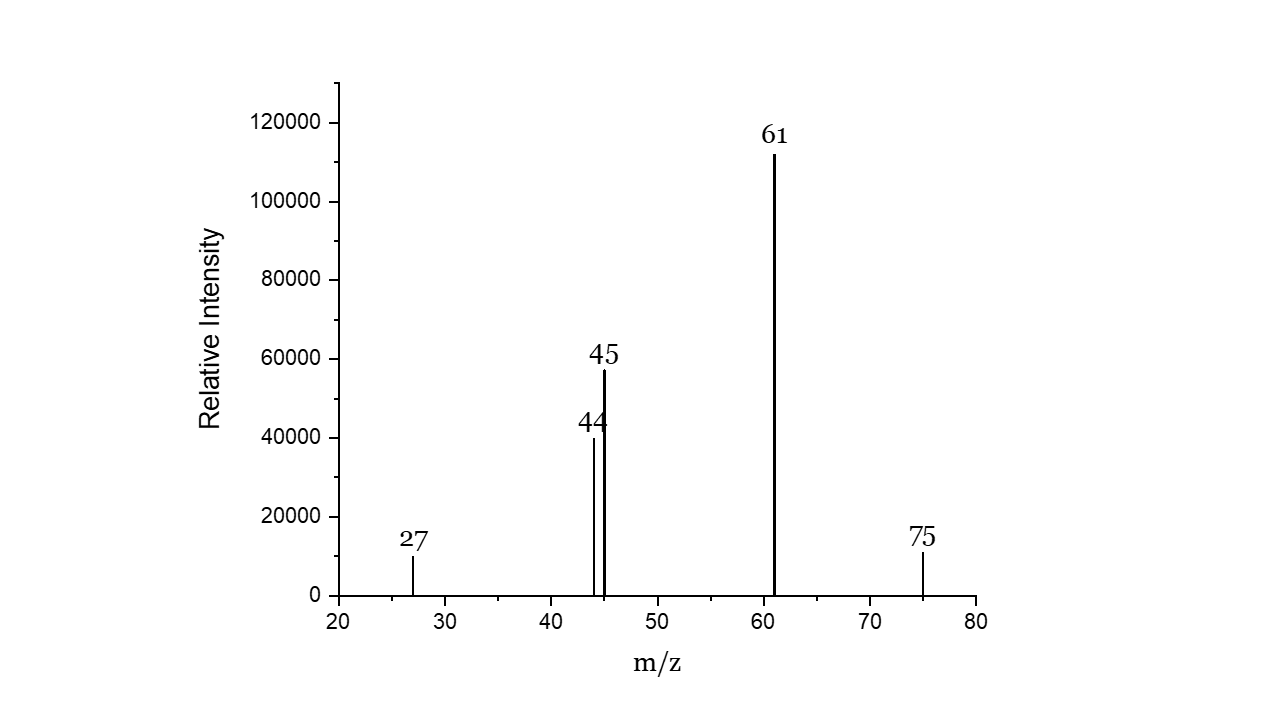

Supplement: S3 Fig — (PNG) [file pone.0349950.s008.png]
